# Improving the Recognition of Equine Affective States

**DOI:** 10.3390/ani9121124

**Published:** 2019-12-11

**Authors:** Catherine Bell, Suzanne Rogers, Julie Taylor, Debbie Busby

**Affiliations:** 1 Equine Behaviour and Training Association, Godalming GU8 6AX, UK; suzanne@learningaboutanimals.co.uk (S.R.); evolutionequine@gmail.com (D.B.); 2 EPONA-TV, 3400 Hillerød, Denmark; julie@epona.tv

**Keywords:** horse, behaviour, welfare, stress, pain, fear

## Abstract

**Simple Summary:**

People commonly fail to recognise the behavioural signs that horses display when they experience pain and fear. Consequently, the distress remains unresolved, reducing the horse’s welfare and having potential safety implications for the handler. In order to investigate the public’s ability to recognise such signs of equine distress, members of equestrian Facebook groups were asked to view and comment on six videos; these videos were selected by the authors on account of their portrayal of horses behaving in a manner suggestive of negative affect. For comparison, responses were also obtained from six equine behaviourists, who identified behaviours suggestive of varying degrees of distress. While respondents successfully recognised behaviour consistent with negative affect in some instances, videos featuring natural horsemanship and bridle-less riding were often wrongly interpreted to be positive experiences for the horses. Despite recognising behaviours indicative of distress in some videos, a minority of respondents nevertheless said they would have been happy for their own horse to be treated similarly. Participant age and experience had little effect on the results; however, responses by people who had selected “clicker training” as their preferred equestrian activity were more closely aligned with those of the equine behaviourists, suggesting that clicker trainers might be more accurate in their recognition of equine distress than other members of the equestrian community. This study can be used to inform the outreach activity of education and welfare organisations, through improved recognition, and subsequent reduction, of equine distress.

**Abstract:**

A key welfare problem for horses is that people commonly fail to recognise, and consequently neglect to resolve, equine behavioural signs of distress, worsening the welfare of the horse and potentially putting the safety of the handler at risk as a result. Members of equestrian Facebook groups were asked to view six videos and assess the horse’s behaviour in each; the authors selected the videos and considered each video to demonstrate behaviour associated with negative affective states. An additional six equine behaviourists also completed the survey as an “expert comparison group” from whom we could define “correct” answers; their responses were consistent with each other and the views of the authors. Although the majority of respondents successfully recognised behaviour indicative of distress in some instances, behaviour associated with negative affective states was commonly missed; videos featuring natural horsemanship and bridle-less riding were particularly interpreted incorrectly to be positive experiences for the horses. Binary logistic regression analysis (72.1% success rate) confirmed that the different video types (ridden dressage, natural horsemanship, in-hand dressage, bridle-less riding, Western reining and behavioural rehabilitation) were strong predictors for obtaining a correct answer (*p* < 0.01) but that experience of equine-ownership was not. Of the equestrian activities preferred by participants, only proponents of clicker training showed an increased likelihood of obtaining the correct answer (*p* = 0.05). Even when behavioural signs suggestive of negative affective states were recognised, a minority of respondents stated that they would be happy for their horse to be treated similarly. In conclusion, behavioural signs of equine distress are poorly recognised; they therefore warrant an increased prominence in education and the outreach activity of welfare organisations, in order to reduce equine suffering.

## 1. Introduction

The failure of equine caregivers to recognise, and subsequently to resolve, expressions of equine stress and pain has been highlighted as a key welfare concern [1]. Lack of education, lack of awareness of behavioural indicators of pain and stress, and misinterpretation of a few such behaviours as “naughty” were thought likely factors. The report [1] concluded that more research into the links between behaviour, stress and pain is required and that more widespread education is needed in order to enhance equine welfare. A preliminary attempt to provide such education has been undertaken by the Equine Behaviour and Training Association (EBTA) [2] in the “Ladder of Fear”, a concept extended from the canine “Ladder of Aggression” [3], but the subject is still commonly omitted from educational programs.

Behavioural indicators of pain and stress are relatively well established in the literature, albeit with a high dependence on physiological information that is unavailable to typical equine owners or handlers. An equine “pain face” and “pain ethogram” have been recognised [4,5,6] and the typical behavioural indicators of stress that have been identified are comparable—muscular tension, triangulated or wide eyes, elimination, tail swishing, ear position, and attempts to flee from frightening stimuli [1,7,8]. A hierarchy of behavioural indicators with increasing stress was found in stabled horses [9], which coupled observable behaviours with physiological measures. Indicators might be subtle, for example eye “wrinkling”, eyelid “twitches” and blink rate [10,11], or more overt such as bucking, head-tossing or rearing for example [12]. Reference [12], a detailed review of studies of behaviours of ridden horses, also concluded that individual differences between horses and inconsistencies amongst researchers (e.g. different behaviours considered and different physiological data obtained) make it difficult to obtain an objective association between behavioural indicators and affective states.

The inability of owners to recognise patterns of behaviours of horses that underpin negative emotional states has been explored by [13] and has been confirmed to have major welfare implications. This is complicated further by the potential for individual horses to be more, or less, overt in their behavioural expression of underlying emotions, and for the effect of training to mask any emotional response that would otherwise be present [14]. Likewise, individual horses cope with stress in different ways [15]. Of particular concern is when a horse appears “calm” or “relaxed” but could equally be described, colloquially, as “switched off” or “shut down”. The potential for learned helplessness—and its causes—in horses was discussed in [16]. Similarly, the likely potential for equine depression has been observed in some ridden horses, which is linked to stereotypical behaviours and episodes of “withdrawal” [17,18]. While it is not clear whether these states are all the one and the same, the likelihood that they exist, coupled with the potential for owners to perceive them as a desirable state such as “relaxed”, is cause for concern. In order to identify behavioural indicators of distress, it is important to recognise that an absence of those indicators does not necessarily equate to an absence of distress.

Identification of behavioural criteria for recognition of negative emotional states is undoubtedly challenging. However, if subtle, early indicators are missed or ignored by the handler, then there is a greater risk that these responses will escalate into more dangerous behaviours (both for human or horse), such as bucking, rearing, kicking, biting, and bolting. A greater number of dangerous behaviours lead to an increased likelihood of punitive action by the handler, mistakenly believing the horse to be recalcitrant, causing a further source of distress to the horse [19]. Therefore, for the sake of equine welfare and human safety, improved communication to the “typical horse owner” as to when horses are suffering from negative affect is needed for example [20].

The aim of this study is to investigate the ability of members of the equestrian community to recognise signs of negative affective state. Without such recognition, the likelihood of such states being alleviated is minimal and becomes a welfare issue.

## 2. Materials and Methods

### 2.1. Respondents

The data used in this study were obtained via a survey that was distributed (in the first instance) in five equine-related Facebook groups (Horse and Hound, Surrey Horse and Pony, Hampshire Horse Riders, Chit Chat and Tack and Happy Horse and Pony), with members based predominantly, but not exclusively, in the U.K. Care was taken to select Facebook groups that were large (>1000 members) and representative of the equestrian community, and no particular training approach was favoured. Some participants opted to share the survey further, into other groups or personal feeds, broadening its audience more widely.

The survey was conducted according to the Code of Ethics and Conduct of the British Psychological Society. Participants were informed of the purpose and contents of the survey, their right to withdraw, data confidentiality and adherence to General Data Protection Regulation; they then provided their informed consent to participate in the study and have their anonymised data published.

### 2.2. Materials

**Figure 1:**
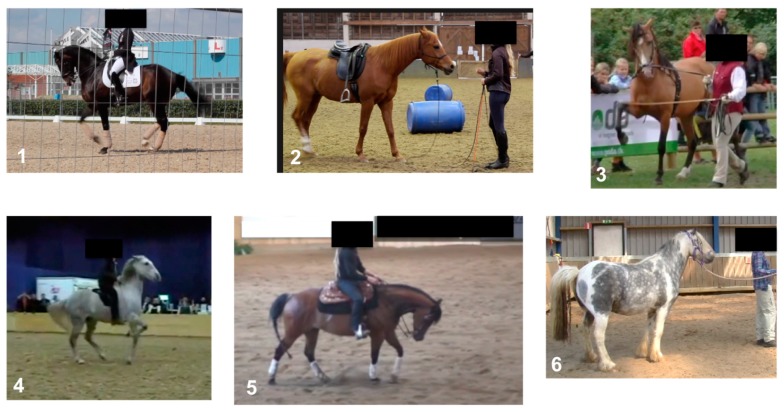
Screen-shots from each of the six videos presented to participants. The horse in each was handled according to a specific equestrian activity—(**1**) ridden dressage, (**2**) natural horsemanship, (**3**) in-hand dressage, (**4**) bridle-less riding, (**5**) Western reining and (**6**) behavioural rehabilitation.

Respondents were shown six video clips with duration of a few seconds to two minutes, in each of which a horse was being handled in a different manner—(1) ridden dressage, (2) natural horsemanship (a training philosophy claiming its origins in observations of wild horse behaviour, for example [21]), (3) in-hand dressage, (4) bridle-less riding, (5) Western reining and (6) behavioural rehabilitation (working to improve the affective state of the horse) (Figure 1). All footage and images were obtained from the archives of EPONA-TV (co-authoring this study), from a body of ongoing investigative, editorial work, which sought to look into the way’s horses are used for sport, leisure and entertainment and the ways in which these uses impact the welfare of the horse. To comply with industry best practice and to respect the privacy of the athletes and performers depicted, all footage was obtained at public events with fully visible camera equipment. The videos were selected on account of the authors recognizing the horses to be demonstrating a variety of behavioural signs of stress, both subtle, such as muscular tension and triangulated eye, and more overt, such as pinned ears and tail swishing. Video 6 (behavioural rehabilitation) was slightly different from the others, in that, although the horse certainly appeared anxious and distressed, the handling was calm, undemanding and was less likely to risk worsening the affective state of the horse. Whilst an understanding of the underlying source of such distress should be paramount in any behavioural rehabilitation work, it was not considered here whether the causes of the behaviours observed in the videos were linked to the training itself, chronic or acute pain, failures of management practices to meet the equid ethogram or the horses’ previous history [22,23]. In particular, no judgment was offered on the handlers, the type of training taking place or the horses’ experiences beyond these clips; instead the focus was on the horses’ behavioural responses that took place during the videos, which might or might not be representative of other occasions.

Demographic information was obtained for each respondent—age, country of residence, preferred equestrian activity (single answer selected from—clicker training, dressage, endurance, eventing, “happy hacker” (i.e., leisure rider), hunting, natural horsemanship, polo, show jumping, Western and “none of the above but enjoy spending time with horses”) and level of experience (multiple answers selected from new (<5 years) horse owner, experienced owner, experienced past owner but currently not a horse-owner, not an owner and professional trainer/instructor/behaviourist/veterinarian or veterinary-nurse/farrier or trimmer/other). Finally, participants were asked to grade on a 4-point Likert scale their self-perceived ability to recognise fear, stress and anxiety in horses, and to describe the behavioural signs that they would normally use to recognise fear and stress. A 4-point scale was chosen to ensure that respondents could be more definite in their response, rather than selecting a mid-point.

Each participant was given a set of 13 possible affective states—angry, anxious, conflicted (defined in the survey as “experiencing two emotions at the same moment in time”), enjoying it, excited, fearful, frustrated, playful, relaxed, stressed, stubborn, submissive, switched off or “resigned”. These affective states might or might not have been experienced by the horse, and the participant was asked to select any number of these options that they recognised in the body language of each horse. The affective states were selected on the basis of being every-day language commonly used by horse owners to describe their horses, correctly or otherwise. Participants were also asked to state on a 3-point Likert scale whether they would be happy for their own horse to be treated in such a manner and, in free text without word limit, to explain their answers. Finally, participants were asked more generally to state, in a free text answer, the behavioural responses that they considered indicative of equine distress.

### 2.3. Procedure

In order to obtain an expert consensus with which a “correct” answer could be defined, the survey was also sent directly to all clinical and accredited equine behaviourists who were listed at the time of the survey on the register held by the Animal Behaviour and Training Council (ABTC). The advantage of using the ABTC register is that those listed are highly qualified and recognised behaviourists in the U.K. and have a variety of backgrounds in terms of education and experience. Therefore, any agreement between them, regarding interpretation of the behaviours demonstrated by the horses in the videos, can be considered to be reasonably accurate. Six of the behaviourists contacted agreed to provide survey responses and consequently became an “expert comparison” group (all qualified with the International Association of Animal Behavior Consultants but with a range of prior experience and qualification) with which the respondent data could be compared. In order to avoid any conflicts of interest, the ABTC behaviourists also authoring this paper were not included in the expert group. While use of the term “behaviourist” is unfortunate, due to behaviourism being concerned only with observable stimulus-response behaviours, it remains accepted terminology in the industry and is used here to be synonymous with “behaviour consultant”.

The data were collected via the Google Forms platform, and prepared for analysis and plotted via a combination of Microsoft Excel and bespoke C-Shell scripts. The quantitative data were analysed using SPSS v. 25 (IBM, Armonk, U.S.A.) for Mac. Initial relationships between variables were assessed using Pearson’s Chi square test of association. Since some pairs of variables had small sample sizes, the significance of any association was confirmed with Fisher’s exact test. Pairs of variables found to have significant associations were explored further using forced entry binary logistic regression analysis.

Since the sample sizes of the experts and participants were too disparate to use for a statistically meaningful comparison, it was necessary to define whether a participant response could be considered “correct” or “incorrect”. In order to find a reasonable balance between robustness and subjectivity, a response was considered “correct” if it met two specific criteria. Criterion 1 required at least one of the responses that 4–6 of the experts ($4\leq N_{Exp}\leq 6$) had selected. Criterion 2 required an absence of all responses selected by zero experts ($N_{Exp}=0)$. All other participant responses were considered “incorrect”. 

## 3. Results

The survey received 185 participant responses in addition to the six expert responses. All experts were located in the U.K., as were 79.5% of the participants. Other participants were located in the U.S.A. (6.5%), Canada (4.9%), other European countries (4.9%), Australia (3.2%), South Africa (0.5%) and Brazil (0.5%). The preferred equestrian activities of participants were numbered as 28.1% dressage, 27.6% “happy hacking”, 10.8% eventing, 10.8% clicker training, 5.4% natural horsemanship, 4.3% endurance, 2.2% Western riding, 2.2% show jumping and 0.5% polo. A further 8.1% said that that they were “none of the above but enjoyed spending time with horses”; for convenience, this group was labelled “others” in later discussion. When asked about their experience with horses, 137 participants claimed to be experienced horse owners, of whom 20 were also professionals within the equine industry; 8 were experienced former owners of whom 2 were also professionals; 19 had owned horses for fewer than 5 years, 2 of whom were professionals; and 5 were not owners at all. A further 16 selected only the ‘professionals’ category. Of the 40 stating that they were professionals within the equine industry, 29 were instructors or trainers, with some also classifying themselves as behaviourists. In response to the question about ability to recognise behavioural expressions of fear, stress and anxiety, 75 claimed “yes, definitely”, 91 “yes, somewhat”, 18 “perhaps/sometimes” and 1 “probably not”.

### 3.1. Expert Responses

When asked generally to state the typical signs that they would use to determine whether a horse was frightened and/or stressed, the experts listed a variety of behavioural responses, of which a subset would likely be shown by an individual horse. While some were overt behaviours, such as rearing or flight, the majority were subtle, such as eye movements, breathing rate or yawning. Some comments reflected a wider understanding of causal factors such as avoidance of tack or changes in social behaviour. While detailed analysis of these qualitative responses is beyond the scope of this paper and will be the subject of a future work, the key point is that the experts were aware of potential early behavioural indications that are likely to reflect the onset of negative affect.

Table 1 shows the responses given by the experts to the videos. The 13 affective states have been listed according to how many experts selected that option: for each video clip, the top line lists the responses selected by 4 or more experts, the bottom line lists unanimously rejected responses and the middle line lists the remaining responses. For example, the experts were unanimous in observing that the horse in video 1 exhibited behaviours associated with stress and did not exhibit behaviours associated with anger, enjoyment, excitement, play, relaxation or submissiveness. For each video, there was unanimity with respect to some video/affect pairs and disagreement for others. However, any contradiction tended to reflect the similar nature of the affective states (e.g., fear vs. anxiety) rather than any fundamental disagreement over the horse’s general experience.

### 3.2. Participant Responses

When asked to list the generic behavioural signs that they would use to determine whether a horse was frightened and/or stressed, many respondents were able to provide a set of subtle behaviours similar to those noted by the experts. However, a greater proportion of overt behaviours were also included, such as bolting, pushiness, vocalising, “foaming at the mouth” or trembling. While these would all have been correct responses, anecdotal experience of working in behavioural practice indicates that earlier and more subtle suggestions of distress would typically also have been present, consistent with the hierarchy of behavioural indicators of stress found in [9].

**Figure 2:**
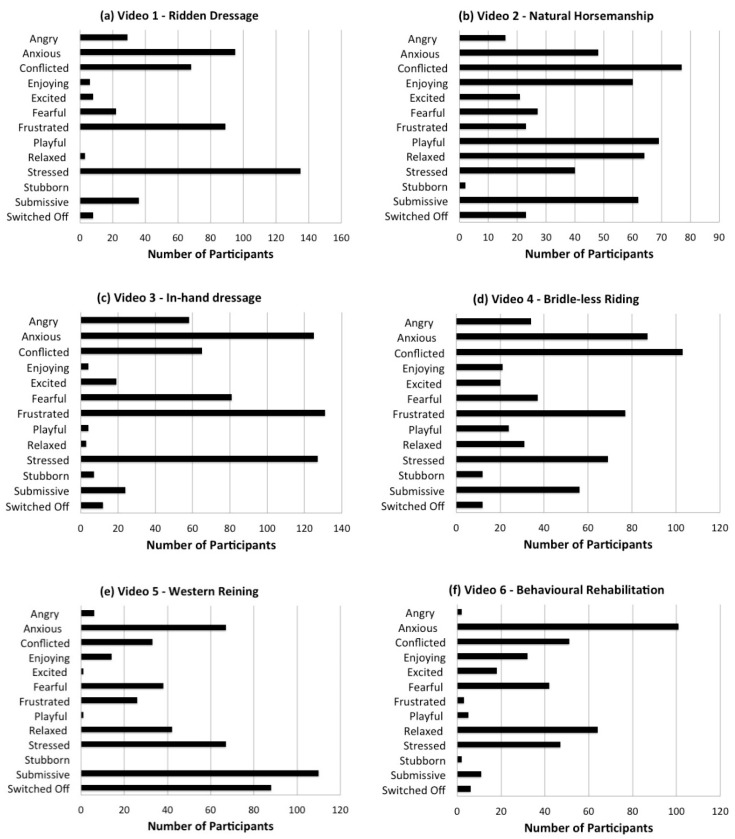
Total numbers of selections of each affective state for each of the 6 videos—(**a**) Video 1: ridden dressage, (**b**) Video 2: natural horsemanship, (**c**) Video 3: in-hand dressage, (**d**) Video 4: bridle-less riding, (**e**) Video 5: Western reining and (**f**) Video 6: behavioural rehabilitation.

All participants selected at least one affective state for each video clip and typically selected more than one. Figure 2 shows all affective state selections for each video. It was clear that the responses included a high number of “anxious”, “conflicted”, “fearful”, “frustrated”, “stressed” and “submissive” selections, in keeping with the experts. However, for some videos there was also a lower but consistent selection of those affective states that were typically not selected by the experts—“angry”, “enjoying it”, “excited”, “playful”, “relaxed” and “stubborn”. Such selections prompted the need for Criterion 2 in defining correct/incorrect responses. For example, a participant selecting “frustrated” and “fearful” for video 1 would be considered correct, whereas “frustrated” and “playful” would be considered incorrect.

**Figure 3:**
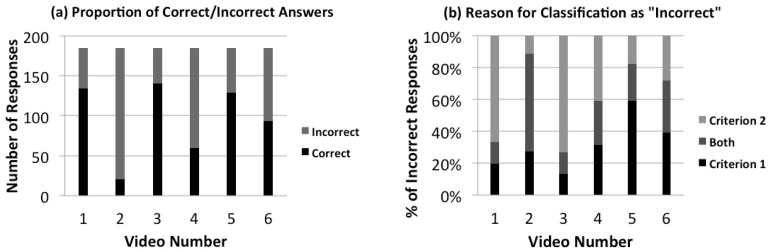
(**a**) Total number of correct responses for each video, (**b**) percentage of incorrect answers in relation to the criterion on which they failed. A response was considered “correct” if it met two specific criteria. Criterion 1 required at least one of the responses that 4–6 of the experts ($4\leq N_{Exp}\leq 6$) had selected. Criterion 2 required an absence of all responses selected by zero experts ($N_{Exp}=0)$. All other participant responses were considered “incorrect”. For each plot, the video numbers correspond to—(1) ridden dressage, (2) natural horsemanship, (3) in-hand dressage, (4) bridle-less riding, (5) Western reining and (6) behavioural rehabilitation.

Figure 3a shows the total numbers of correct and incorrect participant responses as compared to the expert responses. There is a significant difference between the videos ($\chi^{2}\left(5\right)$ = 229.39, *p* < 0.001; Cramer’s V = 0.46, *p* < 0.001) with videos 1, 3 and 5 receiving significantly more correct answers, videos 2 and 4 receiving significantly more incorrect answers; video 6 was almost equal, receiving just one more correct answer than incorrect. Figure 3b shows the total incorrect answers, according to whether they failed on account of Criterion 1, 2 or both. Again, the proportions depended on the video in question, with Criterion 1 prevalent in videos 2 and 5 and Criterion 2 prevalent in videos 1 and 3.

**Figure 4:**
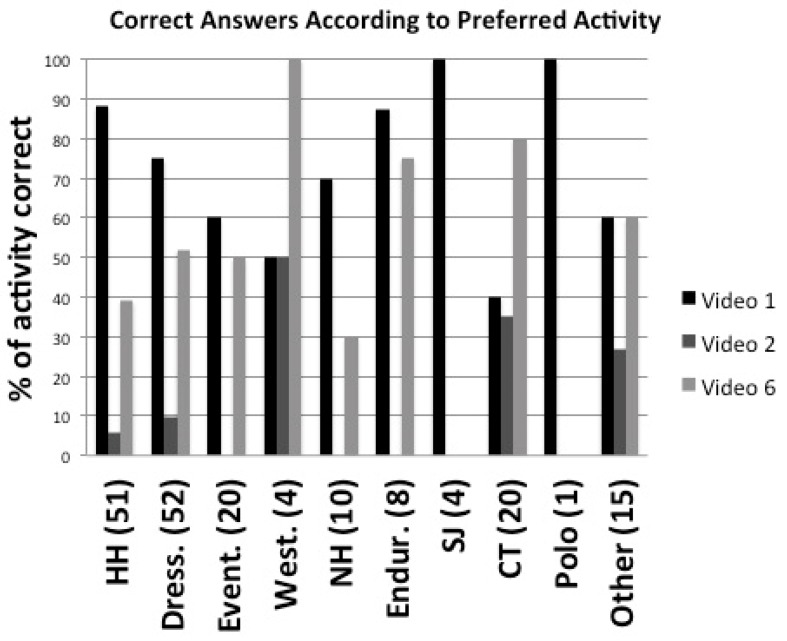
Numbers of correct answers according to preferred activity for videos 1 (ridden dressage), 2 (natural horsemanship), and 6 (behavioural rehabilitation), the three videos for which there was a significant association. Numbers in parentheses indicate total numbers of participants within each activity category.

Pearson Chi-square tests also examined the associations between obtaining a correct answer and the participants’ age, preferred equestrian activity, experience and self-perceived ability to recognise equine distress. Fisher’s exact test was included to account for small sample sizes in some pairings. Videos 3 and 5 showed a significant association between increasing age and correct response, albeit with a relatively small effect size ($\chi^{2}\left(5\right)=12.68$ and 10.11, respectively, *p* < 0.05; Cramer’s V = 0.27 and 0.24 respectively, *p* < 0.05). Videos 1, 2 and 6 showed a significant association and medium effect size between preferred activity and correct response ($\chi^{2}\left(9\right)=22.76,\;21.78,\;19.37$, respectively, *p* < 0.05; Cramer’s V = 0.36, 0.39, 0.33, respectively, *p* < 0.05). These results are illustrated in Figure 4. Ignoring activities with fewer than five participants, video 1 was over-represented by “happy hackers”, dressage riders and endurance riders, video 2 by clicker trainers and “others” and video 6 by clicker trainers, endurance riders and “others”. Videos 3 and 6 showed a significant association between experience and correct response ($\chi^{2}\left(4\right)=16.16,\;12.67$, respectively, *p* < 0.05; Cramer’s V = 0.30, 0.26, respectively, *p* < 0.05), but somewhat erratically, with experienced and new owners obtaining more correct responses in video 3 and professionals scoring higher in video 6. There was no association between correct response and self-reported ability to recognise fear, stress and anxiety for any of the videos.

Binary logistic regression analysis was used to explore the possible association between preferred equestrian activity, participant experience and successful recognition of negative affective states. A model with three predictor variables—Video, Activity and Experience—was used to predict the likelihood of correct answers (*N* = 1,110). The model was successful in predicting 72.1% of the outcomes (*p* < 0.01) compared with the null, intercept-only model. Table 2 lists the categories that had a significant effect on the model. The video categories made a significant contribution to the model, with an increasing likelihood (exp (B) > 1) of correct answers for videos 1, 3 and 5 and a decreasing likelihood (exp (B) < 1) for videos 2 and 4. Past experience and non-ownership made decreasing contributions to the model. Being experienced or a professional did not make a significant contribution to the likelihood of obtaining correct answers. Finally, the only significant (almost, *p* = 0.05) contribution from the activity categories was clicker training, albeit with a very large upper confidence limit.

**Figure 5:**
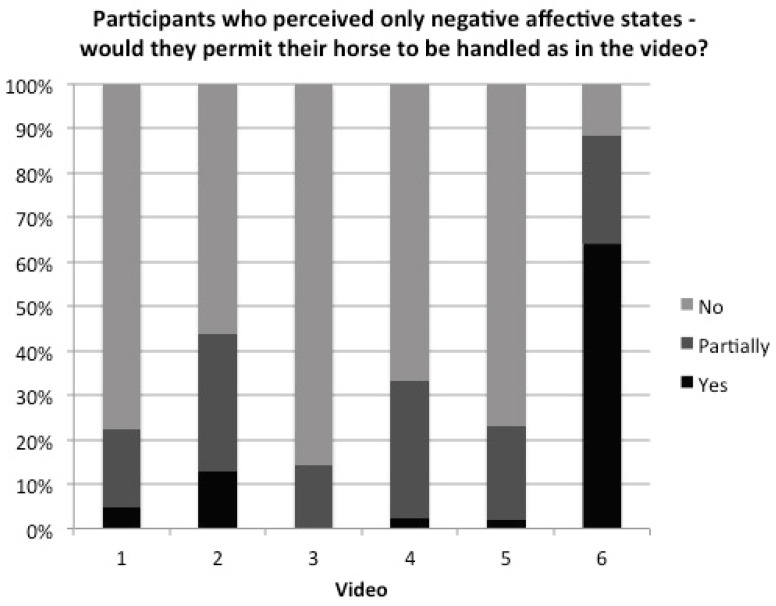
Participants who perceived only negative affective states in each video—(**1**) ridden dressage, (**2**) natural horsemanship, (**3**) in-hand dressage, (**4**) bridle-less riding, (**5**) Western reining and (**6**) behavioural rehabilitation. Black represents those who would, nevertheless, permit their horses to be handled as shown in the video. Dark grey represents those who would do so “partially”. Light grey represents those who would not permit it.

Finally, the participants were asked whether they would be happy for their own horse to be handled in a similar manner as observed in each video. Of particular interest was whether those who believed that less desirable affective states (angry, anxious, conflicted, fearful, frustrated, stressed, stubborn, submissive and switched-off) were present, and that the more pleasurable states (enjoying it, excited, playful and relaxed) were absent, would consider it an acceptable state for the horse to be in during training. The answers are represented in Figure 5; total numbers of participants featured were *n* = 171, 78, 160, 127, 144, 103 for each video, respectively. Black indicates those participants who, despite recognising negative affective states, would still permit their horses to be treated as in the video. Dark grey indicates a middle category for those who would “partially” permit the handling and light grey indicates those who would not. Of the sample of 185, just 6 participants were in the light grey category—i.e. considered the horse to be experiencing negative affective states and that it was unacceptable—for all 6 videos and 30 for videos 1–5. Of the 30, 11 were clicker trainers, compared with 6 claiming no particular activity, 4 dressage, 4 “happy hackers”, 2 eventers, 2 Western riders and 1 natural horsemanship trainer. Of the 30 who considered that the horses in videos 1–5 were experiencing negative affect and that it was unacceptable, 21 classified themselves as current or former experienced owners and 10 were professionals. Note that the complete sample included 40 professionals, 30 of whom, therefore, failed to recognise negative affective states or condoned the handling.

## 4. Discussion

The panel of experts who formed the comparison group gave responses indicating that they believed the horses in all six videos to be demonstrating behavioural signs of stress. Their answers were not identical with one another, but were largely in agreement for the majority of the observations. Any inconsistency was subtle, such as interpreting affective state as “anxious” rather than “stressed”, rather than a fundamentally different qualitative understanding of the affective state. They were unanimous, however, in stating that all horses were experiencing a negative affective state and selected one or more of angry, anxious, conflicted, fearful, frustrated, relaxed, stressed, submissive and switched-off for each horse. They did not consider any of the horses to be experiencing any positive affect, with the exception of just one stating that the horse in video 6 (behavioural rehabilitation) appeared relaxed. None thought any horse showed stubbornness at any point. None of the experts would have been happy for their own horse to be interacted with in the manner shown in videos 1–5; 5 of the 6 experts selected “partially” for video 6 (behavioural rehabilitation), the 6th selected “No”.

The experts’ responses contrast with the participants’ responses and the results of this study support previous findings [1], that equine caregivers do not always recognise the behavioural indicators of stress that are exhibited by horses. While many of the respondents were able to recognise negative affect in at least some of the videos, some simultaneously made contradictory claims for simultaneous positive affect. Videos 2 and 4, featuring natural horsemanship and bridle-less riding were particularly at risk of receiving incorrect responses, suggesting that while participants might be able to detect negative affective states in conventional forms of riding, they are more likely to misinterpret behaviour when part of less traditional (in the U.K.) styles of horsemanship. There is a possibility that some styles of horsemanship foster increased education regarding behaviour; dressage riders, eventers and “happy hackers” obtained more correct responses for video 1, dressage, and clicker trainers were more successful in assessing videos 2 and 6, featuring natural horsemanship and behavioural rehabilitation. Experience appeared to help, but did no guarantee the obtainment of correct answers, and self-reported perception of one’s ability to recognise signs of fear, stress and anxiety had no bearing at all on the likelihood of obtaining correct answers. An interesting note, of the total of 21 respondents who perceived stubbornness in at least one video, 19 were experienced owners (current or former) or professionals, again suggesting that equestrian experience did not guarantee accurate reading of behaviour. Such inconsistency is not unprecedented [24] and adds weight to the call for further formal identification of behavioural indicators of affective states; as well as dissemination of these indicators, and their underlying causes and welfare implications, to the horse-owning public.

Figure 5 represents the respondents who perceived negative affective states in the horses and stated whether or not they would condone such training for their own horse. While it is reassuring from an ethical and welfare perspective that the majority would decline, the statistical minority still represents horse owners (including some professionals) who think it acceptable to train a horse in a manner that is potentially causing distress. This is a mindset that needs to be addressed along with an improved education about behavioural indicators of stress.

Given both the differences in individual horse responses to stressors and the subjectivity of observers, it is perhaps not sufficient to list ethograms of behaviours that might be signs of distress. Good welfare depends on understanding and meeting the physical and emotional needs of the individual horse, as detailed in e.g. [22,25]. The context of the behaviour is also important, in particular whether or not the handler is working effectively to improve the horse’s affective state or merely attempting to complete the human-directed activity. Such a distinction was recognised here in the analysis of video 6 as compared to the others. The horse exhibited similar behaviours yet the session appeared to be focused on improving the horse’s affective state and not attempting to achieve any other goals.

Under the rules of the Fédération Equestre Internationale, dressage judges are expected to recognise and reward “the happy athlete” as a key objective [26]. Of course, the notion of “happy athlete” is meaningless if people fail to recognise the absence of happiness, misinterpret negative welfare states as positive and would sometimes go ahead with training even if they do recognise it to be having a negative impact on the horse. Further research in this area is a key welfare priority, in order to improve recognition of negative affect and incorporate the alleviation of negative affective states into training and education programmes.

### 4.1. Limitations

There were limitations to the design of this study. The survey was more likely to be completed by people interested in behaviour and promotion of the survey on social media groups that were not particularly behaviourally-focused was attempted in order to obtain a sample representative of the equestrian community. However, the survey was shared by friends and supporters of EBTA, increasing the chances of it becoming biased. Future studies could be improved through restricting the survey to (for example) riding clubs or college students rather than relying on the power of social media. In addition, in defining Criteria 1 and 2 it was possible that some responses could be rejected as “incorrect” when they were still possibly correct at a subjective level. For example, anyone labelling the horses in videos 1–5 as “anxious” alone would have been marked as incorrect, arguably erroneously; similarly the distinction between frustrated and angry—the experts selected frustrated but not angry. This effect was mitigated since most respondents selected multiple options for affective states; future studies might be improved if respondents were required to select a set number of affective states instead of allowing this variable to float freely. However, since only one of the experts’ selections was required for a “correct” answer, it was felt that it was already relatively easy for participants to succeed. A final limitation was that “conflicted” and “submissive” were insufficiently defined to be sure that participants were consistent in their use of the terms. “Conflicted” was defined as “experiencing two emotions at the same moment in time” but in some responses the two emotions in question did not seem to be conflicting. Similarly, “submissive” has different connotations to different people; behaviourists commonly regard it negatively, that the horse is overly compliant, however, some respondents clearly regarded it to mean a desirable obedience, again implying a distinction in mindset with potential welfare implications.

### 4.2. Suggestions for Future Study

While this study clearly showed the need for improved recognition of early behavioural indications of negative affective states, the means by which this could be implemented is not straightforward. Clarity in definition of terminology is necessary, as well as caution regarding labels such as “stubborn” or “submissive”, which perhaps reflect a need for a more empathic approach, and investigation into the cause of the behaviour is needed. Furthermore, having clear definitions is of no help to horses unless that information is disseminated more effectively into the horse-owning community; research into the best channels of communication is also key. The expert group responses were almost unanimous and it would be interesting to explore whether agreement could be increased still further, particularly in distinguishing between closely-linked affective states such as fear, stress and anxiety. The study could be improved further if a larger group of experts were consulted, adding weight to the definition of a “correct” answer.

These results also offer the possibility of further associations to explore. What is it about natural horsemanship and bridle-less riding that renders people more likely to misinterpret the horses’ behaviour, when those same people would be more successful if assessing a dressage horse? The Chi square analysis suggested that clicker trainers might have a greater understanding of behaviour than the wider equestrian public and this result remained inconclusive following the binary logistic regression analysis. Further study would be interesting, both to confirm the effect and also with a view to distinguishing whether behaviourally-minded people are attracted to clicker training, or whether the need for detailed understanding of operant conditioning results in a more generalized ability and desire to recognise more subtle behaviours.

## Figures and Tables

**Table 1:** Responses of the 6 experts to the 6 videos included in the survey. Each video shows a horse being handled in a different equestrian activity—(1) ridden dressage, (2) natural horsemanship, (3) in-hand dressage, (4) bridle-less riding, (5) Western reining and (6) behavioural rehabilitation. The second column lists the 13 survey options regarding the affective state of the horses and, in parentheses, the number of experts who selected this option. For each video, the top line lists the responses selected by 4 or more experts, the bottom line lists unanimously rejected responses and the middle line lists the remaining responses.

Video	Expert Response $\left(N_{Exp}\right)$
Video 1	Conflicted (4) Frustrated (4) Stressed (6) Anxious (3) Fearful (2) Submissive (1) Switched-off (1) Angry (0) Enjoying (0) Excited (0) Playful (0) Relaxed (0) Stubborn (0)
Video 2	Stressed (4) Anxious (3) Conflicted (3) Fearful (2) Frustrated (3) Submissive (1) Switched-off (3) Angry (0) Enjoying (0) Excited (0) Playful (0) Relaxed (0) Stubborn (0)
Video 3	Conflicted (4) Fearful (4) Frustrated (4) Stressed (6) Angry (1) Anxious (3) Submissive (1) Enjoying (0) Excited (0) Playful (0) Relaxed (0) Stubborn (0) Switched-off (0)
Video 4	Fearful (5) Stressed (6) Submissive (4) Anxious (3) Conflicted (3) Frustrated (2) Switched-off (2) Angry (0) Enjoying (0) Excited (0) Playful (0) Relaxed (0) Stubborn (0)
Video 5	Fearful (4) Stressed (5) Submissive (4) Switched-off (5) Anxious (3) Conflicted (3) Frustrated (2) Angry (0) Enjoying (0) Excited (0) Playful (0) Relaxed (0) Stubborn (0)
Video 6	Anxious (4) Conflicted (6) Fearful (3) Frustrated (1) Relaxed (1) Stressed (3) Angry (0) Enjoying (0) Excited (0) Playful (0) Stubborn (0) Submissive (0) Switched-off (0)

**Table 2:** Results of the binary logistic regression analysis. A model with three predictor variables—video, activity and experience—was used to predict the likelihood of obtaining a correct answer and was successful in 72.1% of outcomes as compared to the null model. The predictors that had a significant effect on the model are listed; the odds ratio was >1 for increasing contributions to the model and <1 for decreasing contributions to the model.

Predictor Variable	Sig. (*P*)	Odds Ratio (exp (*B*))	95% Conf. Int.
**Ref. (Video 6: behavioural rehab.)**	-	1	-
Video 1 (ridden dressage)	<0.01	3.23	2.05–5.10
Video 2 (natural horsemanship)	<0.01	0.12	0.07–0.21
Video 3 (in-hand dressage)	<0.01	3.25	2.06–5.11
Video 4 (bridle-less riding)	<0.01	0.46	0.30–0.71
Video 5 (Western reining)	<0.010	2.41	1.56–3.73
**Ref. (Experienced)**	-	1	-
Past Experience	<0.01	0.18	0.09–0.35
Not an Owner	0.02	0.37	0.16–0.86
**Ref. (Polo)**	-	1	-
Clicker Training	0.05	9.77	0.97–98.15
